# Effect of Socioeconomic Status and Ethnicity on Glycemic Control in Arab and Jewish Youth with Type 1 Diabetes Mellitus

**DOI:** 10.5041/RMMJ.10350

**Published:** 2018-10-04

**Authors:** Nehama Zuckerman-Levin, Haleema Dabaja-Younis, Elemy Ameer, Michal Cohen, Yasmin Maor, Naim Shehadeh

**Affiliations:** 1 Pediatric Diabetes Unit, Endocrinology, Diabetes, and Metabolism Institute, Rambam Health Care Campus, Haifa, Israel; 2 The Ruth & Bruce Rappaport Faculty of Medicine, Technion–Israel Institute of Technology, Haifa, Israel; 3 Infectious Disease Unit, Wolfson Medical Center, Holon, Israel; 4 The Sackler Faculty of Medicine, Tel Aviv University, Tel Aviv, Israel

**Keywords:** Ethnicity, glycemic control, socioeconomic status, type 1 diabetes mellitus

## Abstract

**Objectives:**

Research and theory suggests that socioeconomic status may affect diabetes control. We investigated the effect of socioeconomic status and ethnicity on glycated hemoglobin (HbA1c) in Arab and Jewish children with type 1 diabetes mellitus in northern Israel.

**Methods:**

Data were collected from medical records of 80 Arab and 119 Jewish children attending a pediatric diabetes clinic in a tertiary health care center. Multivariate regression analysis was used to assess factors independently affecting HbA1c level.

**Results:**

Mean age was 12.9±4.7 years. Arab families had more children compared to Jewish families (3.7±1.5 versus 2.9±1.2, respectively, *P*=0.0007). Academic education was significantly less common in Arab families (25% versus 66.2%, respectively, *P*=0.0001). Income of Jewish parents was significantly higher compared to that of Arab parents (7,868±2,018 versus 5,129±906 NIS/month, respectively, *P*=0.0001). Mean age at diagnosis of diabetes was 9.6±4.6 years and disease duration was 3.4±2.3 years in both groups. Half of Arab and Jewish children were treated with multiple insulin injections and half with insulin pumps. Mean number of self-glucose testing/day was higher in Jewish children than in Arab children (4.7±2.5 versus 4.0±1.5, respectively, *P*=0.033). Mean HbA1c was above recommendations, 9.5% (12.6 mmol/L) in Arab children and 8.7% (11.3 mmol/L) in Jewish children (*P*=0.004). In multivariate analysis, disease duration (*P*=0.010) and ethnicity (*P*=0.034 for Arabs versus Jews) were independently associated with HbA1c.

**Conclusions:**

Both Arab and Jewish children failed to meet HbA1c goals, but this effect was significantly greater for Arabs. Ethnicity remained a predictor of failure even following adjustment for potential confounders.

## INTRODUCTION

Type 1 diabetes mellitus (T1DM) is a common chronic disease in children and adolescents. It is caused by insulin deficiency as a result of destruction of the insulin-producing beta cells in the pancreas. It accounts for 75%–80% of newly diagnosed diabetes in patients younger than 18 years.^1^–^4^ The cause of beta cell destruction seems to be a state of chronic autoimmunity and inflammation against these cells. Destruction of the beta cells causes a relative or absolute insulin-deficient state, and as a result hyperglycemia occurs. Genetics, immune state and trait, infectious morbidity, and environmental factors are all thought to contribute to the development of T1DM.^5^,^6^ Due to the fact that children need to constantly inject insulin and monitor their blood glucose levels, involvement of parents, lifestyle adaptation, and good communication with the treating physicians, nurses, and dieticians are essential.^7^ The responsibility for attaining the appropriate glycated hemoglobin (HbA1c) goal rests upon the children and their caregivers, with close and continuing support from the clinic.^8^ Reaching and maintaining the metabolic goals is of particular importance as studies have shown that attaining them decreases microvascular complications.^9^ Thus, the control of T1DM is influenced by factors related to the patients but also to their caregivers. These factors include, among others, patients’ age, duration of illness, socioeconomic status, level of education of the parents, and the degree of parental responsibility and involvement.^10^–^14^ Studies have demonstrated the impact of socioeconomic status on the level of HbA1c both in adults and children.^14^–^17^ The effect of race per se on metabolic control is less clear. In some studies an effect of race was demonstrated, perhaps relating to genetic factors, while other studies failed to demonstrate an added effect of race over other sociodemographic factors.^18^,^19^

Documentation and registration of T1DM incidence among Israeli children and youth (aged 0–18 years) began in Israel in 1996. Data relying on the registry show that the incidence of T1DM in Israel increased from 8 cases per 100,000 persons in 1997 to 12.5 cases per 100,000 people in 2009 and to 13.9 cases per 100,000 people in 2011. It seems that the rise in the incidence of T1DM cases occurs both in Jewish and Arab children and adolescents, but the rise is more pronounced in the Arab population.^20^,^21^ The reason for this changing epidemiology is not clear, and it is not known whether similar factors affect glucose control in Arab and Jewish children and adolescents. A recent study in the southern part of Israel demonstrated ethnic differences in glycemic control between the Jewish and Bedouin populations.^22^

The purpose of this study was to assess factors affecting metabolic control in Arab and Jewish youth with T1DM and to assess whether ethnicity affects glucose control beyond the impact of sociodemographic factors. The study was performed in a pediatric diabetes clinic in a tertiary health center in the North of Israel, the Rambam Medical Center.

## METHODS

### Study Design and Subjects

We conducted a retrospective analysis of a convenience sample of T1DM patients who regularly attended the Pediatric Diabetes Clinic at Rambam Health Care Campus, between May 2013 to May 2014. The hospital’s internal review board approved the study. In the clinic, children, adolescents, and young adults in the age range of infancy to 35 years old are treated.

To be included in the analysis, patients had to be between the ages of 1 and 30 years old at the time defined above and to fulfill the American Diabetes Association (ADA) criteria of T1DM diagnosis.^23^ Additionally, they needed to have a follow-up period of more than six months at the clinic, and to have completed at least two regular follow-up visits during the defined period.

Ninety-five percent of the patients had evidence of autoimmune diabetes with positive antibody screen for anti-GAD, anti-IA-2, or Insulin Ab (one or more).

Patient data were collected from patient files that fulfill the inclusion criteria without recruitment bias.

### Data Collection

Data were summarized from patients’ medical records in a tabulated manner. Information included the following: *sociodemographic parameters*: age, gender, ethnicity, parents’ income, number of children in the family; *co-morbidity*: celiac disease, hypothyroidism, hypertension, and hyperlipidemia;* disease presentation*: age at diagnosis, keto-acidosis at presentation, family history of diabetes; *diabetes management*: insulin type, mode of insulin administration, adherence to treatment, number of daily self-glucose measurements and continuous glucose monitoring; *acute complications*: events of metabolic acidosis, microvascular complications; and *laboratory data*: HbA1c, lipid profile.

The primary outcome was metabolic control as reflected by mean HbA1c levels in the past year. Our definition for target HbA1c value was <7.5%, as defined by the American Diabetes association (ADA).^24^ Thus, patients were categorized into two groups depending on their HbA1c levels: poorly controlled patients with HbA1c ≥7.5%, and well controlled patients with HbA1c <7.5%.

### Statistical Methods

Descriptive statistics of patient data was performed and expressed as means and standard deviations (SD) for continuous variables and as number and percentage for dichotomous variables. Statistical significance was set at *P*<0.05. Variables were compared using Student’s *t* test and chi-square. Univariate analyses were performed, with the dependent variable being HbA1c. Thereafter, multivariate analysis was performed; dependent variables were selected as candidate variables if *P*<0.2 on the univariate analysis. All analyses were conducted using standard statistical software (SPSS version 22, Inc., Chicago, IL).

## RESULTS

### Sociodemographic Characteristics

Altogether 199 T1DM patients were included in the study. Of these, 80 (40.2%) were Arabs, and 119 (59.8%) were Jews. Patients’ characteristics are presented in Table 1. Mean age was 12.3±4 years for Arab children and 13.3±5 years for Jewish children; 64% and 54% were males, in Arab and Jewish samples, respectively. Arab families had significantly more children compared to Jewish families (3.7±1.5 versus 2.9±1.2, respectively, *P*=0.0007).

**Table 1:** Patient Characteristics.

Characteristic	Arabs (*n*=80)	Jews (*n*=119)	*P*
Sociodemographic characteristics			
Age (years)	12.34±4.28	13.30±5.06	0.162
Gender (males)	51 (63.8%)	64 (53.8%)	0.163
Number of children in the family	3.73±1.49	2.87±1.23	<0.001
Fathers with academic education	9 (15.0%)	39 (60.9%)	<0.001
Mothers with academic education	13 (21.7%)	37 (55.2%)	<0.001
At least one parent with academic education	15 (25.0%)	45 (66.2%)	<0.001
Parents’ income (NIS)	5129±918	7869±2028	<0.001
Risk factors for diabetes and co-morbidity			
Diabetes in first-degree relatives	7 (8.8%)	12 (10.1%)	0.754
Diabetes in first- and second-degree relatives	53 (66.3%)	47 (39.5%)	<0.001
Body mass index (BMI)	20.03±4.21	20.01±3.49	0.980
Hypothyroidism and celiac disease	6 (7.5%)	6 (5.0%)	0.475
Disease parameters			
Age at diagnosis of diabetes (years)	8.98±4.31	10.08±4.84	0.102
Duration of T1DM (years)	3.49±1.90	3.44±2.63	0.900
Ketoacidosis at diagnosis of T1DM	26 (33.3%)	33 (27.7%)	0.401
Use of multiple daily injections	39 (48.8%)	55 (46.2%)	0.726
Use of insulin pump	40 (51.2%)	64 (53.8%)	0.601
Use of continuous glucose monitoring	11 (13.8%)	24 (20.2%)	0.244
Number of glucose self-tests per day	4.07±1.59	5.00±3.51	0.013
Outcome			
HbA1c (% [mmol/L])	9.54% [12.6]±1.98% [0.6]	8.70% [11.3]±1.71% [0.1]	<0.002
HbA1c >7.5% (>9.3 mmol/L)	69 (89.6%)	88 (76.5%)	0.021

Data presented as average±SD or *n* (%) for all patients.

NIS, Israeli new shekel.

Jewish parents had more years of academic education compared to Arab parents, as can be seen in Table 1, for both fathers and mothers. Only in 25% of Arab families did at least one parent have an academic education, whereas among Jewish families 66.2% had at least one parent with an academic education (*P*=0.0001). Income was significantly higher in Jewish parents compared to Arab parents (7,868±2,018 versus 5,129±906 NIS per month, respectively, *P*=0.0001).

### Risk Factors for Diabetes and Co-morbidity

In both Arab and Jewish families about 9%–10% of first-degree relatives had diabetes, a figure comparable to that usually reported.^25^,^26^ The total number of first- and second-degree relatives with diabetes (either type 1 or type 2) was higher in Arab families compared to Jewish (66.3% versus 39.5%, respectively, *P*=0.002).

Among other factors, mean body mass index was 20±3.8 kg/m^2^ in both groups. Hypertension was found in only two patients, both Arabs. Hyperlipidemia was documented in 42% of both Arabs and Jews.

There was a low rate of other autoimmune diseases such as hypothyroidism and celiac disease in both groups.

### Disease Parameters

Mean age of diagnosis of diabetes was 9.1±4.3 years in the Arab population and 10±4.8 years in the Jewish population (*P*=not significant). Mean duration of disease was 3.4±1.9 years in both groups. Diabetic ketoacidosis (DKA) was the presenting symptom of diabetes in ~30% of patients in both ethnic groups, in contrast to the higher incidence of DKA at presentation described in other studies from Israel_._^21^,^22^,^27^ In both ethnic groups, about half of the children were treated with multiple daily injections of insulin per day, and about half were treated with a continuous insulin infusion pump. Continuous glucose monitoring was used by 13% of Arab children and 20% of Jewish children. The mean number of self-glucose testing per day was significantly higher in Jewish children compared to Arab children (4.7±2.5 versus 4.0±1.5, respectively, *P*=0.033).

### Glycemic Control

Mean HbA1c was well above the recommended target HbA1c in both Arab and Jewish children, yet levels were even higher for Arab children compared to Jewish children (9.5%±2% [12.6 mmol/L] versus 8.70%±1.6% [11.3 mmol/L], *P*= 0.004, respectively). The percentage of metabolically uncontrolled children with an HbA1c higher than 7.5% (9.4 mmol/L) was significantly higher in Arab children (89.6% versus 76.5% in Jewish children, *P*=0.021). Family size did not affect glycemic control in either Arab or Jewish children. Mean HbA1c hemoglobin increases with age in the Arab patients (*r*=0.26, *P*=0.022) but not in the Jewish patients (*r*=0.06, *P*=0.489).

Additional DKA after diagnosis was observed in 10.4% of all patients. Ethnicity did not change the occurrence. Patients with at least one episode of additional DKA had worse glycemic control (*P*=0.008).

Studying age at diagnosis and disease duration as contributors to glycemic control, with HbA1c as continuous or dichotomous variable, demonstrated higher HbA1c with younger age at diagnosis and prolonged duration of disease, in both ethnic groups.

No correlation was found between glycemic control and family history of diabetes in either Arab or Jewish patients.

Factors significantly and directly associated with HbA1c levels in univariate analysis were ethnic origin (Arabs > Jews, *P*=0.002), parents’ academic education (*P*=0.003), income level of parents (*r*= −0.15, *P*=0.044), and duration of disease (*r*=0.18, *P*=0.015) (Table 2). In a multivariate regression analysis, where the dependent variable was mean HbA1c and the independent variables in the model were ethnic origin, parents’ academic education, income level of parents, and duration of disease, the only parameter that was independently associated with HbA1c level was parents’ academic education (β= −0.20, *P*=0.042) (Table 3). When omitting parents’ education, both disease duration (β=0.17, *P*=0.018) and ethnicity (β= −0.21, *P*=0.027) remained independently associated with HbA1c levels (Table 4).

**Table 2:** Univariate Analysis of Factors Significantly Associated with HbA1c.

	B	SE	β	*P*	95% Confidence Interval
Ethnicity	−0.84	0.27	−0.22	0.002	−1.37, −0.31
Age	0.05	0.03	0.12	0.105	−0.01, 0.10
Gender (males)	−0.42	0.27	−0.11	0.127	−0.95, 0.12
Number of children in the family	0.08	0.11	0.06	0.480	−0.14, 0.30
Parents with academic education	−0.97	0.33	−0.26	0.003	−1.62, −0.33
Parents’ income	−0.01	0.01	−0.15	0.044	−0.001, −0.01
Age at diagnosis	0.01	0.03	0.03	0.680	−0.05, 0.07
BMI	0.05	0.04	0.11	0.147	−0.02, 0.12
Duration of T1DM	0.14	0.06	0.18	0.015	0.03, 0.25

**Table 3:** Factors Independently Associated with HbA1c.

	B	SE	β	*P*	95% Confidence Interval
Ethnicity	−0.59	0.45	−0.16	0.188	−1.47, 0.29
Parents with academic education	−0.75	0.37	−0.20	0.040	−1.48, −0.03
Parents’ income	0.01	0.01	0.03	0.791	−0.01, 0.01
Duration of T1DM	0.13	0.07	0.16	0.068	−0.01, 0.27

Adjusted *R^2^*=0.08, *P*=0.008.

Multiple regressions analysis, *n*=120.

**Table 4:** Factors Independently Associated with HbA1c without Parents’ Education.

	B	SE	β	*P*	95% Confidence Interval
Ethnicity	−0.80	0.36	−0.2	0.027	−1.51, −0.09
Parents’ income	−0.01	0.01	−0.01	0.898	−0.01, 0.01

Duration of T1DM	0.14	0.06	0.17	0.018	0.02, 0.25

Adjusted *R^2^*=0.06, *P*=0.002.

Multiple regressions analysis, *n*=178.

Studying disease parameters by multivariate regression analysis where the dependent variable was mean HbA1c and the independent variables were ethnicity, parent education, income level, DKA at diagnosis, length of illness, mode of treatment (pump versus injections), and mode of monitoring (self-monitoring versus continuous glucose monitoring), the only parameter that was independently associated with HbA1c level was self-monitoring (β= −0.24, *P*=0.012).

### Morbidity

Our cohort of young patients and short duration of disease did not demonstrate a high percentage of microvascular morbidity. Nephropathy was the only morbidity that was found in 4% of patients.

## DISCUSSION

Type 1 diabetes mellitus is a chronic disease that requires excellence in compliance, close and multi-disciplinary follow-up, and a thorough understanding of the disease and its complications by the patients and their caregivers. Parents play an important role in attaining treatment goals. Our results demonstrate that most Arab and Jewish children and adolescents treated in a tertiary center in northern Israel did not reach the target glycemic goal of HbA1c less than 7.5%. Furthermore, HbA1c levels were significantly higher in Arab children and adolescents compared to their Jewish counterparts. This finding is surprising as both groups had very similar disease parameters such as age, age at diagnosis, additional autoimmune diseases, DKA at diagnosis, duration of T1DM, BMI, and mode of treatment. The main differences that we could identify in this cohort relates primarily to socio-demographic parameters. The percentage of Arab parents with academic degrees was significantly lower than that of Jewish parents. Moreover, Arab parents’ income was significantly lower compared to that of Jewish parents. Studies have shown that health literacy is important for better understanding of diabetes goals^28^ and that a lower education level and lower income are associated with increased mortality and higher HbA1c levels in children with T1DM.^16^,^28^–^30^ In contrast to studies examining the effect of socioeconomic factors that were carried out elsewhere, we must emphasize that health insurance coverage for T1DM patients is the same for the whole population in Israel, thus socio-economical difference had no impact on the quality of medical care and medical supplies provided to our patients.

We also demonstrated that Arab families had significantly more children compared to Jewish families. This may affect the time and energy Arab parents can devote to their diagnosed child and thus may affect HbA1c levels. Even though our results did not demonstrate that family size affects glycemic control, the association of ethnic origin and worse HbA1c outcomes may be mediated by family milieu, unawareness of the disease and its treatment, as well as parental education level and income, and not necessarily by unstudied genetic factors.

Our study demonstrated that Arab families had more first- and second-degree relatives with diabetes compared to Jewish families. Most of these patients were diagnosed with type 2 diabetes mellitus (T2DM). It is well known that T2DM is more related to genetic factors as well as to social and cultural factors such as diet, weight, and physical activity.^31^ Rates of type 2 diabetes in Arabs in Israel are twice the rate reported in the Jewish population.^32^ Studying the genetic profile across the Arab world suggested that the high prevalence of T1DM in Arabs may be also related to genetic factors.^33^

Another factor affecting the attainment of target HbA1c was the performance of self-tests: fewer glucose self-tests were performed by Arab children compared to Jewish children. In both ethnic groups, achievement of improved glycemic control was related to better self-monitoring. It has been demonstrated that children who perform more blood glucose tests achieve lower HbA1c levels compared to children who perform fewer self-tests.^34^,^35^

In multivariate analyses, the main factor independently associated with HbA1c level was parents’ academic level. When omitting this variable, which enabled us to increase the sample due to fewer missing values, ethnicity and duration of T1DM emerged as factors independently associated with HbA1c. This again reinforces the importance of socioeconomic status on the outcome of diabetes that may be camouflaged by ethnicity.

This study has several limitations. Data were collected retrospectively from patients’ files. Thus, information was not complete and was not collected systematically from all patients in real time. As we explored only well-established potential factors of metabolic control in children with diabetes, it is possible that potential cultural factors (such as acceptance and perception of the disease in different cultures) that were not evaluated may have had an impact on the outcomes.

To conclude, in our cohort of T1DM patients better glycemic control was achieved in patients with short duration of disease, who were treated by multiple injections and monitored by multiple self-monitoring blood tests. This study demonstrated, in accordance with other studies,^22^,^36^ that most children and adolescents do not attain the target HbA1c. The effects were more concerning for Arab children, with significantly higher HbA1c levels. Our results suggest that the main drivers of this disparity are a lower education level of Arab parents and a lower income in Arab families. Other factors such as culture and genetics may also play a role. Our conclusions led us to investigate educational programs designed to help T1DM patients understand the theoretical diabetes management and its biochemical basis. Preliminary unpublished data from that research demonstrate that education and increased knowledge improve the understanding of diabetes self-management and enhance regulation of blood glucose. The effect of some socioeconomic parameters such as family income is reduced when knowledge is increased. Urgent intervention is warranted for both the Arab and Jewish population to improve glycemic control.

## Abbreviations

DKA: diabetic ketoacidosis
HbA1c: Hemoglobin A1c (glycated hemoglobin)
T1DM: Type 1 diabetes mellitus
